# Perspective: next generation isotope-aided methods for protein NMR spectroscopy

**DOI:** 10.1007/s10858-018-0198-x

**Published:** 2018-06-22

**Authors:** Masatsune Kainosho, Yohei Miyanoiri, Tsutomu Terauchi, Mitsuhiro Takeda

**Affiliations:** 1 Graduate School of Science, Tokyo Metropolitan University, 1-1 Minami-ohsawa, Hachioji, Tokyo, 192-0397 Japan; 2 Structural Biology Research Center, Graduate School of Science, Nagoya University, Furo-cho, Chikusa-ku, Nagoya, 464-8602 Japan; 3 Research Center for State-of-the-Art Functional Protein Analysis, Institute for Protein Research, Osaka University, 3-2 Yamadaoka, Suita, Osaka 565-0871 Japan; 4 SI Innovation Center, Taiyo Nippon Sanso Corp., 2008-2 Wada, Tama-city, Tokyo 206-0001 Japan; 5 Department of Structural BioImaging, Faculty of Life Sciences, Kumamoto University, 5-1, Oe-honmachi, Chuo-ku, Kumamoto, 862-0973 Japan

**Keywords:** Isotope-aided NMR method for larger proteins, Stereo-array isotope labeling (SAIL), TROSY by isotope labeling, SAIL aromatic ^13^CH TROSY, SAIL aliphatic ^13^CH TROSY, ^1^H-direct observation at ultrahigh-field

## Abstract

In this perspective, we describe our efforts to innovate the current isotope-aided NMR methodology to investigate biologically important large proteins and protein complexes, for which only limited structural information could be obtained by conventional NMR approaches. At the present time, it is widely believed that only backbone amide and methyl signals are amenable for investigating such difficult targets. Therefore, our primary mission is to disseminate our novel knowledge within the biological NMR community; specifically, that any type of NMR signals other than methyl and amide groups can be obtained, even for quite large proteins, by optimizing the transverse relaxation properties by isotope labeling methods. The idea of “TROSY by isotope labeling” has been cultivated through our endeavors aiming to improve the *original* stereo-array isotope labeling (SAIL) method (Kainosho et al., Nature 440:52–57, 2006). The SAIL TROSY methods subsequently culminated in the successful observations of individual NMR signals for the side-chain aliphatic and aromatic ^13^CH groups in large proteins, as exemplified by the 82 kDa single domain protein, malate synthase G. Meanwhile, the expected role of NMR spectroscopy in the emerging integrative structural biology has been rapidly shifting, from structure determination to the acquisition of biologically relevant structural dynamics, which are poorly accessible by X-ray crystallography or cryo-electron microscopy. Therefore, the newly accessible NMR probes, in addition to the methyl and amide signals, will open up a new horizon for investigating difficult protein targets, such as membrane proteins and supramolecular complexes, by NMR spectroscopy. We briefly introduce our latest results, showing that the protons attached to ^12^C-atoms give profoundly narrow ^1^H-NMR signals even for large proteins, by isolating them from the other protons using the selective deuteration. The direct ^1^H observation methods exhibit the highest sensitivities, as compared to heteronuclear multidimensional spectroscopy, in which the ^1^H-signals are acquired via the spin-coupled ^13^C- and/or ^15^N-nuclei. Although the selective deuteration method was launched a half century ago, as the first milestone in the following prosperous history of isotope-aided NMR methods, our results strongly imply that the low-dimensional ^1^H-direct observation NMR methods should be revitalized in the coming era, featuring ultrahigh-field spectrometers beyond 1 GHz.

## Introduction

The NMR spectra of larger proteins are characterized by numerous overlapped signals, which are severely broadened by dipolar interactions between nearby protons. Therefore, it is practically impossible to observe all of the individual NMR signals for quite large proteins. Conceptually, however, it may not be necessary to gather all of the NMR signals for studying protein structures and dynamics, since the amino acid side-chains contain somewhat redundant structural information. For example, if we could deuterate either one of the prochiral methyls or methylene protons stereo-specifically, then the missing information could be compensated more than adequately by the remaining geminal counterpart signals, which have unambiguous stereospecific assignments. The stereo-array isotope labeling (SAIL) method systematically creates such situations for all of the amino acid residues in proteins, by trimming off the redundant information by isotope-labeling. The *original* SAIL amino acids preserve the through-bond ^13^C–^13^C and ^13^C–^15^N connectivity paths for the sake of the backbone and side-chain sequential assignments, and completely eliminate the ambiguities of the stereo-specific assignments for all of the prochiral groups (Kainosho et al. 2006, 2018; Kainosho and Güntert 2009). Although the overall non-exchangeable proton density in a SAIL protein, which is exclusively composed of SAIL amino acids, becomes reduced to 50–60% of that of the fully protonated protein, the remaining protonated sites are perfectly untouched. It is quite important to emphasize that, even though the level of deuteration is as high as 60%, only a single isotopomer exists for a SAIL protein. The terminology “stereo-array isotope labeling” actually seeks to highlight this striking feature of this new isotope-labeling approach.

The significantly decreased overall proton density for a SAIL protein mitigates the spin diffusion without sacrificing the signal intensities of the remaining protonated sites, and thus facilitates the acquisition of accurate inter-proton NOEs for larger proteins. By virtue of these distinctive features of the SAIL method, facile and accurate structure determinations of proteins as large as 40–50 kDa have become feasible. Obviously, there is a practical size limit for protein structural determinations even by the SAIL method, since spectral analyses become incorrigibly cumbersome for extraordinarily large proteins. Fortunately, the other methods, such as X-ray crystallography and cryo-electron microscopy, have significantly advanced during the past decades. These methods are more efficient and widely applicable for proteins regardless of their sizes; therefore, the structure determinations of quite large proteins may no longer be the primary role for NMR spectroscopy. Instead, in the contemporary paradigm of integrative structural biology, NMR spectroscopy is envisaged as the main technique to study protein structures and dynamics. This information would be most efficiently obtained through the NMR probe signals belonging to the functionally important regions of proteins, and thus is crucial to understand their biological functions.

Although the structural probes are presently limited to the TROSY methyl and backbone amide signals for larger proteins (Pervushin 2000; Tugarinov et al. 2003; Tugarinov and Kay 2003), in this article we show that any type of NMR signals other than those from methyl and amide groups; *i.e*., aromatic and aliphatic CH groups, can also be observed equally well even for the 82 kDa single domain protein, malate synthase G (MSG), by optimizing their transverse relaxation properties by appropriate isotope labeling. We purport that these newly accessible NMR signals will provide unprecedented opportunities for studying the structures and dynamics of larger proteins and protein complexes.

## Transverse relaxation optimized SAIL (SAIL TROSY) approach

Tugarinov and Kay successfully observed narrow ^1^H–^13^C HMQC signals for the methyl groups of the Ile, Leu and Val (ILV) residues in deuterated MSG, in which the ILV methyls were selectively labeled with ^13^CH_3_ by using metabolic precursors (Tugarinov et al. 2003; Tugarinov and Kay 2004). Although the ILV residues represent only 22% of the 723 residues of MSG, a well-defined structural model was obtained by combining all of the information obtained by the ILV methyls and the backbone ^15^NH signals. Since the methyl signals of the Ala, Thr and Met residues could also be observed, the methyl-containing amino acid residues thus represented as much as 40% of the MSG protein (Isaacson et al. 2007; Godoy-Ruiz et al. 2010). A commonly held belief is that the rapid rotation around the *pseudo* threefold axis is a prerequisite to generate narrow ^1^H–^13^C HMQC signals, as derived from the theoretical consideration (Tugarinov et al. 2003). This breakthrough technology, which is often referred to as “methyl TROSY”, has been widely used for studying large proteins together with ^15^NH TROSY (Pervushin et al. 1997; Pervushin 2000). If similar considerations are also valid for the methylene and methine groups of the amino acid side-chains, then they would rarely provide narrow NMR signals. Fortunately, this is not the case for the methylene and methine groups. Although they cannot rotate rapidly on their own, the aliphatic ^13^CH groups generate narrow line-widths by improving their transverse relaxation properties by the optimal isotope labeling strategy; i.e., the “TROSY by isotope labeling”. We also observed the narrow aromatic ring ^13^CH signals for large proteins by the aromatic TROSY method (Pervushin et al. 1998), with further optimization of the transverse relaxation properties of the original SAIL aromatic amino acids by isotope labeling. Although the aromatic amino acid residues, Phe, Tyr, Trp and His, represent only 10% of the total residues in MSG, the structural information about the bulky side-chain rings is extremely important for filling in the gaps between the methyl groups and the backbone amides.

## Observation of the aromatic ring ^13^CH signals

Pervushin et al. showed that the aromatic ring ^13^C signals exhibit the TROSY effect, as a consequence of the cross relaxation effect between the ^13^C chemical shift anisotropy (CSA) and the dipolar–dipolar (DD) relaxation due to the directly bonded proton (Pervushin et al. 1998). Since they used [U–^13^C,^15^N]-proteins, the DD-CSA cross-relaxation effect was far from the optimum, due to the complex scalar and dipolar spin coupling networks. It is interesting to mention that according to the theoretical prediction, the TROSY effect on the ^13^C T_2_ is most efficient at around 600 MHz (Pervushin et al. 1998). This issue was recently revisited by Takeuchi et al. in the context of the optimal field strengths for direct observations of ^15^N and ^13^C NMR (Takeuchi et al. 2016). Takeuchi et al. claimed that the maximum ^1^H-detected ^15^NH TROSY and ^13^C-detected aromatic ^13^CH TROSY should be around 1.5 GHz and 900 MHz, respectively. We actually found that the ^1^H-detected aromatic 2D ^13^CH TROSY sensitivity increases substantially, by increasing the field strengths in the range of 600–900 MHz. This may depend on the performance of the NMR equipment, but it is also affected by the drastically simplified spin-networks implemented by the SAIL aromatic amino acids (Torizawa et al. 2005; Takeda et al. 2010; Miyanoiri et al. 2011, 2012; Yang et al. 2015).

**Fig. 1:**
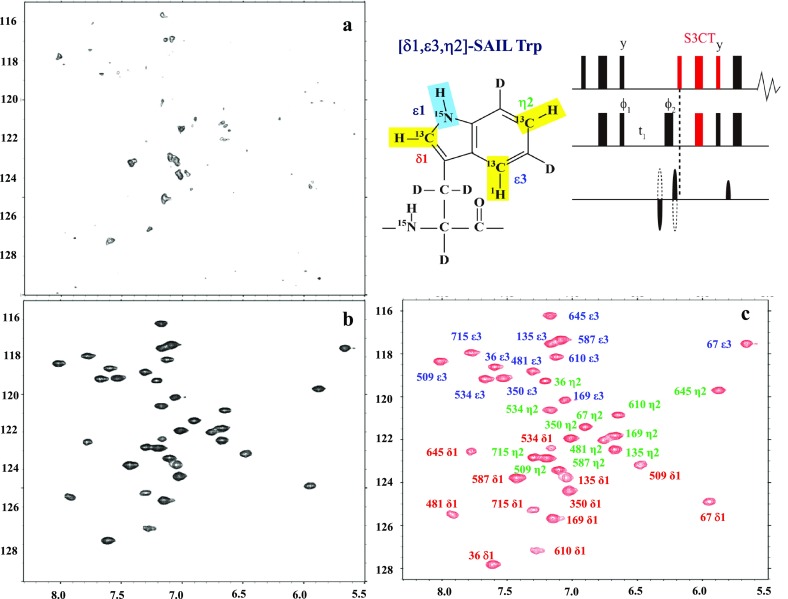
Aromatic regions of the 800 MHz 2D ^1^H–^13^C correlation spectra of the MSG selectively labeled with [δ_1_,ε_3_,η_2_]-SAIL Trp, and otherwise uniformly labeled with deuteron and ^15^N. Sample: 0.3 mM in 20 mM sodium phosphate buffer (pH 7.1), containing 20 mM MgCl_2_, 5 mM DTT, and 5% D_2_O. NMR: NS = 128, d1 = 1.5 s, TD = 1024 × 256, 310  K. The total experimental time for each spectrum was ~ 7 h with a cryogenic probe. **a** F1-decoupled HSQC; **b** Aromatic ^13^CH TROSY; **c** Aromatic ^13^CH TROSY with signal assignment

We prepared MSGs labeled with either ζ-SAIL Phe or [δ_1_, ε_3_, η_2_]-SAIL Trp by the conventional *Escherichia coli* cellular overexpression system, using deuterated M9 medium, and found that the labeling rates were higher than 90% for both amino acids, even with small amounts of the SAIL Phe or SAIL Trp (~ 10 mg/L). The 2D aromatic ^13^CH correlation spectra were measured at 800 MHz for a 0.3 mM solution under various conditions, to define the optimal acquisition parameters. The F1-decoupled HSQC showed virtually no signals even after ~ 7 h, since the anti-TROSY components broaden the F1-decoupled signals beyond detection (Fig. 1a). However, the ^1^H–^13^C TROSY HSQC, using the S3CT pulse sequence (Sørensen et al. 1997), gave 36 beautiful cross peaks for the δ_1_, ε_3_, an η_2_ CHs for the 12 Trp residues of MSG within the same duration (Fig. 1b). All of the signals are well dispersed within the large chemical shift ranges for ^13^C (~ 12 ppm) and ^1^H (~ 2.5 ppm). The assignments of the signals were completed as follows. The δ_1_-CH signals were identified by correlating the δ_1_-^13^CH to the ε_3_-^15^NH through the ^1^H–^15^Nε–^13^Cδ–^1^H connectivity, and the ε_3_-signals were identified by labeling MSG with the ε_3_-dueterated [δ_1_, ε_3_, η_2_]-SAIL Trp. The sequential assignments for the cross peaks were firmly established by a series of single amino acid replacements of each Trp residue with Phe (Fig. 1c).

**Fig. 2:**
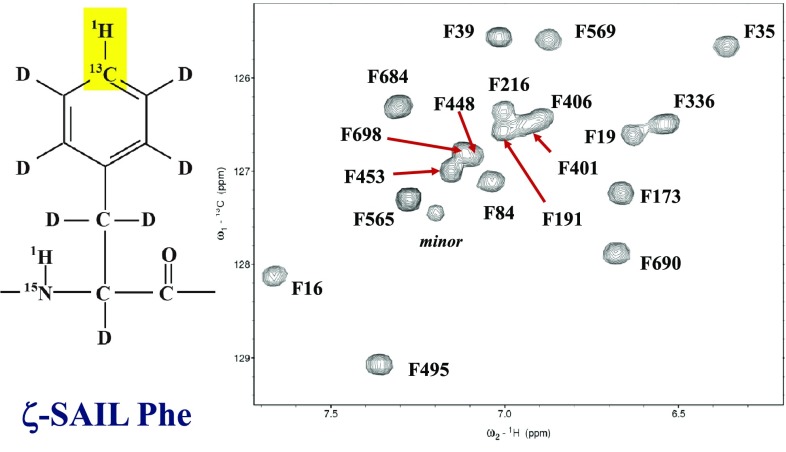
Aromatic region of the 900 MHz 2D aromatic ^13^CH TROSY spectrum of the MSG selectively labeled with ζ-SAIL Phe, and otherwise uniformly labeled with deuteron and ^15^N. Sample: 0.15 mM in 20 mM sodium phosphate buffer (pH 7.1), containing 20 mM MgCl_2_, 5 mM DTT, and 5% D_2_O. NMR: NS = 64, d1 = 2 s, TD = 1024 × 128, 310 K. The total experimental time was ~ 9 h with a cryogenic probe

We also illustrate the ^1^H–^13^C TROSY HSQC spectrum obtained for the MSG selectively labeled with ζ-SAIL Phe. Although we used a 0.15 mM H_2_O solution in this case, 19 well resolved signals for the 19 Phe residues were obtained at 900 MHz within ~ 9 h (Fig. 2). The sequential assignment shown in the figure was initially completed by a series of single amino acid mutations for each residue. The assignment strategy using a series of single amino acid replacements has been successfully used for the assignments of methyl signals in large proteins (Amero et al. 2011). In the case of bulky aromatic rings, we should be especially careful not to cause serious structural perturbations by mutations, and thus we used either Tyr or Leu to replace each Phe residue. The other aromatic ^13^CH signals of Tyr and His in MSG were also observed using transverse relaxation optimized SAIL amino acids. Many of the assignments for the aromatic protons by the amino acid replacement method have been further confirmed by the ^1^H–^1^H NOEs between the ILV methyl protons, for which the sequential assignments were firmly established (Tugarinov et al. 2003).

## Observation of aliphatic ^13^CH signals other than methyls

Since the backbone amides, side-chain methyls, and aromatic rings comprise the considerable portion of a protein, their narrow NMR signals provide valuable information about the structures and dynamics of larger proteins. However, this information is not completely sufficient for the detailed analysis of the conformational dynamics of the biologically important side-chain moieties, which could be manifested most directly by their aliphatic methylene and methine signals. If we recollect the theoretical considerations for methyl TROSY (Tugarinov et al. 2003), the aliphatic methylene and methine groups are not likely to generate narrow NMR signals, since they are not amenable to the line-narrowing effects by rapid rotation, DD-CSA interference, or interference between auto- and cross-correlated ^1^H–^1^H dipolar relaxation. In contrast to these pessimistic expectations, we proved that very narrow methylene and methine ^13^CH signals can be obtained for the isolated ^13^CH pairs, implemented by suppressing the major ^1^H–^1^H and ^13^C–^13^C scalar and dipolar coupling pathways using appropriate isotope labeling.

**Fig. 3:**
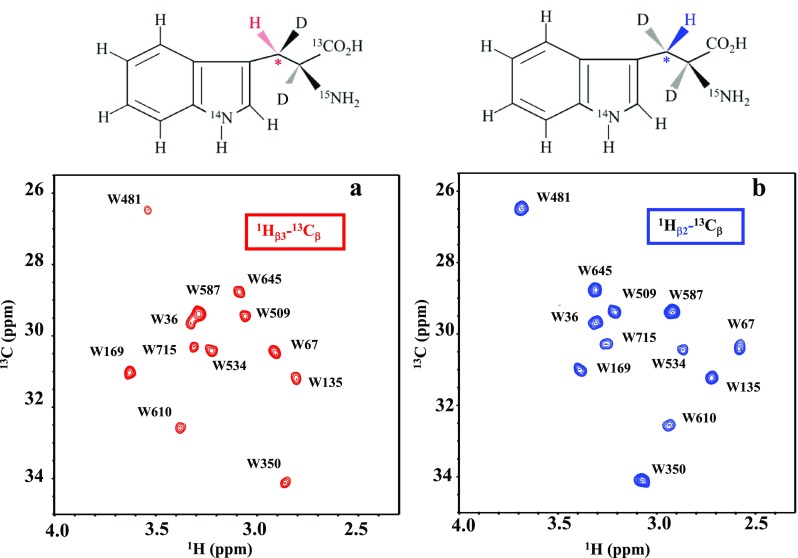
2D Aliphatic ^13^CH HSQC spectra of MSGs selectively labeled with **a** [β-^13^C; β_2_-D]-Trp or **b** [β-^13^C; β_3_-D]-Trp, and otherwise fully deuterated. Sample: 0.12 mM in 20 mM sodium phosphate buffer (pH 7.1), containing 20 mM MgCl_2_, 5 mM DTT, and 5% D_2_O. NMR: d1 = 4 s, TD = 1024 × 128, 310 K

We synthesized two prototypes of tryptophan customized for observing the β_3_H and β_2_H signals, in which the ^13^C_β_ has no directly bonded ^13^C and either one of the prochiral protons is stereo-specifically deuterated (Fig. 3). Even though the indole ring remains protonated, we successfully observed all 12 of the ^13^CH pairs for each of the MSG proteins expressed in deuterated M9 medium containing a small amount (10 mg/L) of either [β-^13^C; β_2_-D]-Trp (Fig. 3a) or [β-^13^C; β_3_-D]-Trp (Fig. 3b). The sequential assignments of the ^13^C_β_ were readily established by adapting the ^13^C_β_ chemical shift data obtained by the 4D NMR analysis of [U–^13^C,^15^N, D]-MSG (Tugarinov et al. 2002). Their data fit almost completely with our ^13^C_β_ data, by accounting for the small differences due to the isotope shifts. These assignments were further confirmed by the intra- and inter-residue NOEs and by the single amino acid replacements, as described above.

## Structure information obtained by the aromatic and aliphatic ^13^CH signals

It is now apparent that all of the aromatic and aliphatic ^13^CH signals could be observed and assigned for proteins as large as 80 kDa. In a preliminary effort to estimate the molecular weight limit for observing aromatic and aliphatic ^13^CH signals by “the SAIL by isotope labeling” method, we successfully observed the aromatic TROSY ^13^CH signals for an 880 kDa *Thermus thermophilus* chaperonin (GroES–EL complex). Therefore, these newly accessible NMR probes provide unprecedented opportunities to apply a variety of NMR techniques, which have been exclusively used for small and medium size proteins, to extremely large proteins and protein complexes. Although some of the local structure information acquired from the aromatic and aliphatic ^13^CH signals may seem too trivial for studying supramolecular complexes, it is quite important to emphasize that such information had never been obtained for extremely large proteins. In the following, we show two examples.

### The side-chain conformation of Trp residues

**Fig. 4:**
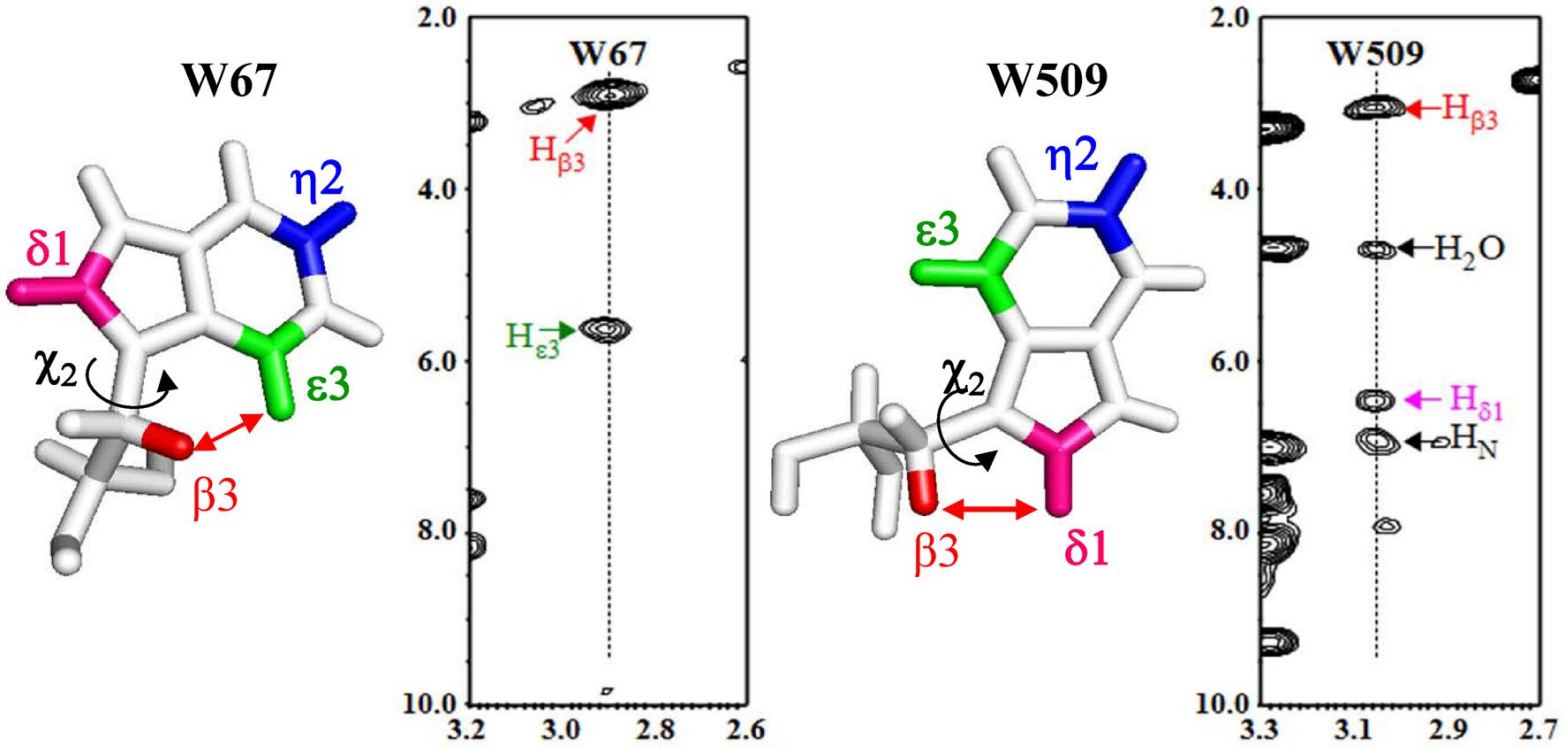
900 MHz 3D ^13^C-edited NOESY-HSQC spectrum of the MSG selectively labeled with β_2_ deuterated SAIL-Trp, and otherwise fully deuterated. The two strips for the W67 and W509 residues are shown. The χ^2^ angles and the β_3_-δ_1_, ε3 hydrogen distances in the crystal (PDB 1D8C): W67, χ^2^ = + 103º, β_3_-δ_1_ 3.8 Å, β_3_-ε_3_ 2.7 Å; W509, χ^2^= − 102º, β_3_-δ_1_ 2.6 Å, β_3_-ε_3_ 4.3 Å. Sample: 0.25 mM, in 20 mM sodium phosphate buffer (pH 7.1), containing 20 mM MgCl_2_, 5 mM DTT, and 1% D_2_O. NMR: NS = 32, mixing time = 200 ms, TD = 2048 × 20 × 128, d1 = 4 s, 310 K

The side-chain conformation of a Trp residue in a protein can be defined by two rotation angles, χ^1^ and χ^2^. The χ^2^ angle, which represents the rotation angle around the C_β_–C_γ_ bond, can be estimated by the intra-residue NOEs between the side-chain β-H and the indole ring δ_1_- and ε_3_-H. The SAIL Trp residue was quite useful to observe the intra-residue NOEs relevant to the χ^2^ angles, as shown for the 7 Trp residues in the 12 kDa cMyb-R2R3 domain (Miyanoiri et al. 2011). In this small protein, we could determine the χ^1^ and χ^2^ rotamers using the relevant intra-residue NOEs, without the deuteration of the residues other than SAIL Trp. The same approach could be applied to determine the three χ^2^ rotamers; i.e., ± 90° and 0°, for the 12 Trp residues in the 82 kDa MSG, although we had to fully deuterate MSG in order to maximize the ^13^CH TROSY effect. In Fig. 4, we show two strips of the 3D ^13^C-edited NOESY-HSQC for the MSG selectively labeled with the “β_2_-deuterated” SAIL-Trp, to illustrate the intra-residue NOEs for the W67 and W509 residues. According to the crystalline state, the χ^2^ angles of W67 and W509 are + 103° and − 102°, respectively. The χ^2^ angle difference between these two residues results in the inter-proton distances between H_β3_ and δ_1_- and ε_3_-H. The NOEs were observed only for the β_3_-ε_3_ (2.7 Å) and β_3_-δ_1_ (2.6 Å), for W67 and W509, respectively, indicating that the χ^2^ angles determined in the solution and the crystal are the same. Although the χ^2^ angles of W67 and W509 can be roughly estimated as + 90° (± 30º) and − 90° (± 30º) by NMR, the side-chain conformations of the aromatic amino acids play a crucial role in the local structures around them.

### The relative orientation between aromatic rings

**Fig. 5:**
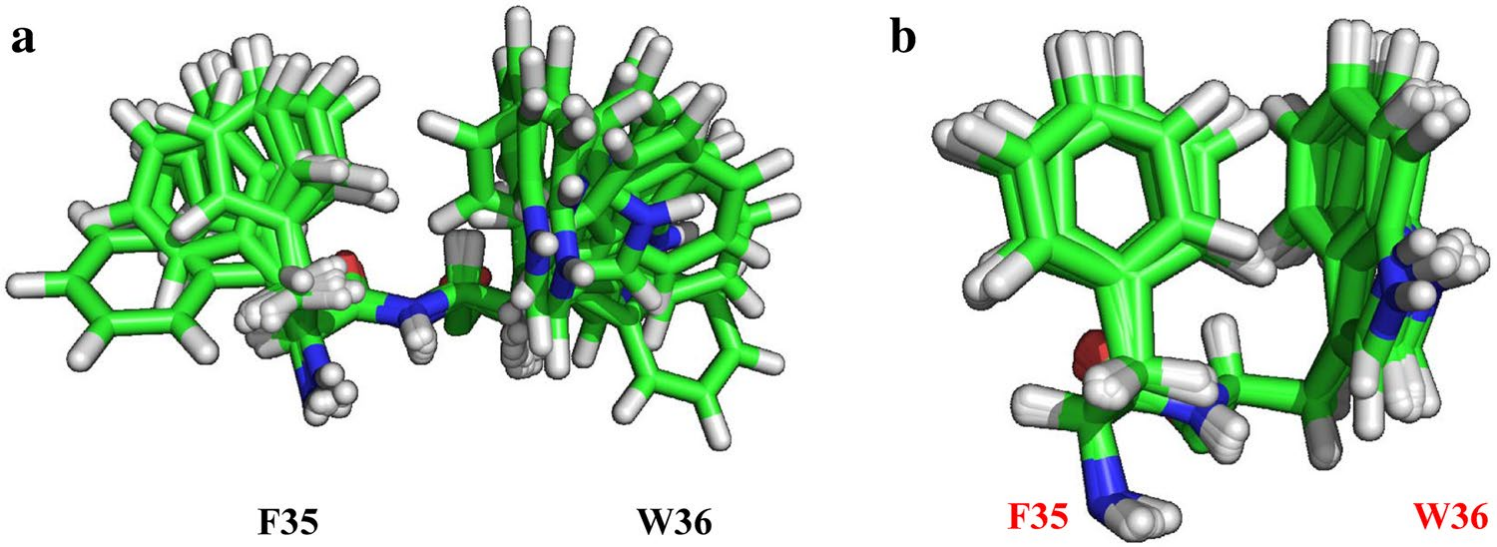
Effect of the implementation of the additional NOE constraints acquired for the aromatic and aliphatic methylene protons on the NMR structure of MSG. Structural calculations including NOE constraints for **a** ILV methyls and backbone amides; **b** ILV methyls, backbone amides, and aromatic ring protons of Phe, Tyr, and Trp residues. Only the relative orientation between F35 and W36 is illustrated, although the overall structure was substantially refined by including the NOEs involving aromatic and aliphatic protons (details will be published elsewhere)

The inter-residue NOEs associated with the aromatic ring protons are especially important, to acquire the local structural information around the bulky aromatic rings. We illustrate the most obvious case for determining the relative orientations of two spatially close aromatic rings; namely, the aromatic rings of F35 and W36. As shown in Fig. 5a, the relative orientation between the two aromatic rings cannot be defined in the NMR structures calculated based on the NOEs for the ILV methyls and backbone amides. Obviously, the NOE data set has no distance constraints for the relative orientation between the two rings. However, the overall NMR structures calculated based on the NOE data sets including the aromatic ring protons of 19 Phe, 18 Tyr and 12 Trp residues (a total of 49 residues) converged much better, especially in terms of the relative orientation between the aromatic rings, as illustrated in Fig. 5b, as did the structures around the ILV methyls and aromatic rings. We are still collecting NOEs and hope to prove that the NMR structures of MSG ultimately converged as robustly as those for small proteins.

## A next generation labeling strategy: Low-dimensional ^1^H-direct observation spectroscopy at ultra-high fields over 1 GHz

We described above a breakthrough isotope labeling strategy to observe aromatic and aliphatic ^13^CH signals for extremely large proteins. The idea of transverse relaxation spectroscopy (TROSY) by isotope labeling, which is the heart of this new technology, emerged from the original SAIL method, which was developed more than a decade ago (Kainosho et al. 2006, 2018). During the past decade, however, the major role of NMR spectroscopy in structural biology has dramatically changed from structure determinations to structure dynamics studies of biologically interesting targets, such as membrane proteins and supramolecular complexes. Therefore, we hope that newly accessible NMR probes, besides methyls and backbone amides, for difficult targets will contribute to maintain NMR spectroscopy as the major player in the coming era of integrative structural biology. In the future, ultrahigh-field NMR spectrometers will be available to the biological NMR community and scientists will keenly desire access to them. Obviously, such machines will be unaffordable for individual laboratories, and thus they should be shared. The machine time allocated to each laboratory will be quite limited, and therefore it should be used most efficiently. This is somewhat an entirely new situation for the NMR community, since the major NMR laboratories always have their own high-field equipment. Therefore, we definitely need much more sensitive NMR methods than ever explored.

The advanced isotope-aided methods developed so far are based on the indirect ^1^H-detection via spin-coupled heteronuclei, such as ^13^C and ^15^N. Although there are many innovative techniques to shorten the measurement time, unavoidably most of the ^1^H-NMR magnetization is lost during the coherent transfer processes in heteronuclear multidimensional spectroscopy. Recently, Takeuchi et al. mentioned the same problem and suggested that amide ^15^N-detected TROSY and aromatic ^13^C-detected TROSY would be most sensitive at 1.15 GHz and 900 MHz, respectively (Takeuchi et al. 2016). These nuclei have much lower gyromagnetic ratios (γ) as compared to ^1^H, and thus their inherent NMR sensitivities are much lower than ^1^H. On the other hand, due to their low γ values, they give narrower line-widths, because of that they are less sensitive to the dipolar broadening from the nearby ^1^H signals. The effect may be largely offset by the remaining CSA for the amide ^15^N and aromatic ^13^C, and thus it is where the direct ^1^H-detection method comes in.

**Fig. 6:**
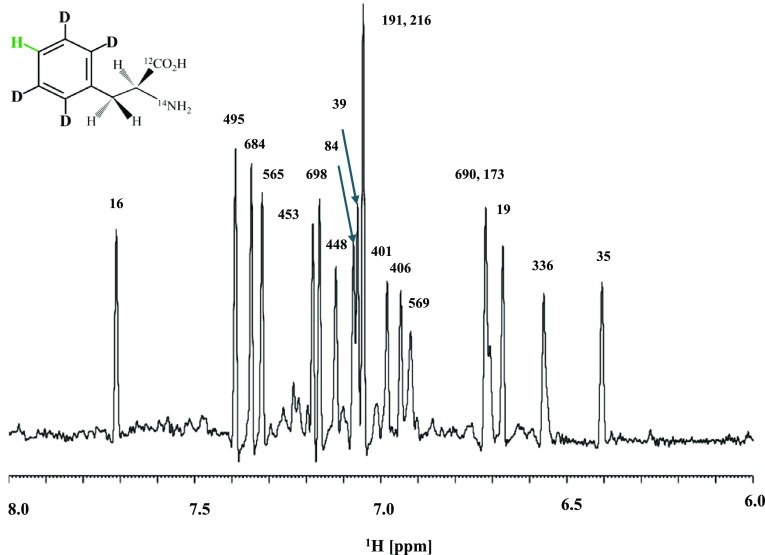
900 MHz 1D ^1^H-NMR spectrum of [U-D;δ,ε-D_4_-Phe]-MSG. Sample: 0.2 mM in 20 mM deuterated sodium phosphate buffer (pH ~ 7), containing 20 mM MgCl_2_ and 5 mM DTT. NMR: d1 = 30 s, NS = 512, 310 K. The total experimental time was ~ 4.5 h. The signal assignments were transferred directly from the 2D aromatic ^13^CH TROSY spectrum shown in Fig. 2

Proton (^1^H) is the most sensitive nucleus other than tritium (^3^T), and has very little CSA. Therefore, if there is no other ^1^H nearby, the isolated ^1^H attached to ^12^C should have the highest sensitivity. Actually, the method, called “selective deuteration”, was developed a half century ago, as the first isotope-aided NMR method to observe the isolated ^1^H of aromatic amino acids embedded a deuterated background (Markley et al. 1968). We revisited this old method to observe the ^1^H^12^C_ζ_ signals of Phe residues in the MSG labeled with [δ,ε-D_4_]-Phe, with an otherwise fully deuterated background (Fig. 6). Surprisingly, but somewhat expectedly, extremely narrow and well dispersed ^1^H_ζ_ signals were observed at 900 MHz. All of the signals were readily assigned just by transferring the ^13^CH TROSY signal assignment, shown in Fig. 2. There were no other signals in this region, since the amide protons were fully deuterated. The ^1^H_ζ_ of the Phe residue in MSG has virtually no other protons within a few Å, and the observed line-widths are about 5–6 Hz, a value about one order of magnitude smaller than the ^1^H line-widths estimated from the TROSY ^13^CH signals at 900 MHz. The sensitivity is reasonably high, since the spectrum was obtained with a 0.2 mM solution with 512 transients. A long T_1_ value for the isolated ^1^H^12^C was an anticipated problem, and thus we used a relatively long relaxation delay time (d1) of 30 s to acquire the 512 transients, and the total experimental time was ~ 4.5 h. The long T_1_ problem would be largely improved by incorporating the ILV methyls simultaneously, as they served as an efficient heat sink.

The 1D ^1^H-NMR is especially useful for the aromatic ring protons, since there are only amide protons in the same chemical shift region, which can be completely eliminated by deuterium substitution in D_2_O. However, low-dimensional ^1^H-direct detection NMR spectroscopy may not be restricted to the aromatic ring protons, and should be explored as the next generation isotope-aided method in the ultrahigh-field NMR era. Since any type of proton has a relatively small CSA, the present approach of “selective protonation” should work better at higher field strengths, if we could overcome the T_1_ versus T_2_ problem by controlling the relaxation paths.
